# Inducible Expression of both *ermB* and *ermT* Conferred High Macrolide Resistance in *Streptococcus gallolyticus* subsp. *pasteurianus* Isolates in China

**DOI:** 10.3390/ijms17101599

**Published:** 2016-09-22

**Authors:** Meixia Li, Chao Cai, Juan Chen, Changwei Cheng, Guofu Cheng, Xueying Hu, Cuiping Liu

**Affiliations:** College of Veterinary Medicine, Huazhong Agricultural University, 1st Shizishan Street, Wuhan 430070, China; mislimeixia@126.com (M.L.); cccaochao@yeah.net (C.C.); cj879503625@163.com (J.C.); 13027120595@163.com (C.C.); chengguofu@mail.hzau.edu.cn (G.C.); hxying@mail.hzau.edu.cn (X.H.)

**Keywords:** *Streptococcus gallolyticus* subsp. *pasteurianus*, *ermB*, *ermT*, macrolide

## Abstract

*Streptococcus gallolyticus* subsp. *pasteurianus* is an under-recognized pathogen and zoonotic agent causing opportunistic infections in humans. Despite increasing recognition of this subspecies as a cause for human infectious diseases, limited information is known about its antibiotic resistance mechanism. In this study, we aim to identify the molecular mechanism underlying the high macrolide resistance of six *S. gallolyticus* subsp. *pasteurianus* isolates from dead ducklings collected in several natural outbreaks in China during 2010–2013. All isolates exhibited multi-drug resistance including high macrolide resistance (MIC ≥ 1024 mg/L for erythromycin, and 512 mg/L for clarithromycin). Efflux-encoding *mefA* and *mefE* genes were not detectable in these isolates. The presence of 23S rRNA mutations in specific isolates did not significantly change macrolide MICs. No nucleotide substitutions were found in genes encoding ribosomal proteins L4 or L22. The *ermB* and *ermT* genes were found in the genomes of all isolates. These two genes were acquired independently in one highly virulent isolate AL101002, and clustered with Tn*916* and IS*1216*, respectively. The expression of both *ermB* and *ermT* in all isolates was erythromycin inducible and yielded comparable macrolide MICs in all six isolates. Taken together, inducible expression of both *ermB* and *ermT* conferred high macrolide resistance in these *S. gallolyticus* subsp. *pasterianus* isolates. Our findings reveal new macrolide resistance features in *S. gallolyticus* subsp. *pasteurianus* by both *ermB* and *ermT*.

## 1. Introduction

*Streptococcus gallolyticus* subsp. *pasteurianus* is a member of the group D streptococci. It was previously named *Streptococcus bovis* biotype II/2, based on its ability to decarboxylate gallate, to produce β-galactosidase and β-glucuronidase, and inability to ferment mannitol or produce trehalose [1,2]. However, owing to frequent ambiguity in *S. bovis* taxonomy following these phenotypic characterizations, *S. bovis* biotype II/2 was renamed to *S. gallolyticus* subsp. *pasteurianus* according to its genetic properties [3,4], and became one of the three subspecies of *S. gallolyticus* along with *S. gallolyticus* subsp. *gallolyticus* and *S. gallolyticus* subsp. *macedonicus* [4].

The ambiguity of *S. bovis* taxonomy led to limited clinical study on *S. gallolyticus* subsp. *pasteurianus*, and it is thus considered as an underreported opportunistic pathogen in both animals and humans. It can cause meningitis in ducklings and septicemia in pigeons, goslings and turkey poults, leading to high mortality in these poultries [5,6,7,8]. In humans, it is increasingly recognized as a cause of infant meningitis and bacteremia [9,10,11,12], and 58.7% of mortality rate has been observed with one-year neonates relating to this subspecies [13]. In adults, it is linked to a wide spectrum of diseases, including meningitis [14], endocarditis [15,16,17], splenic abscess [18], biliary and urinary tract infection [15,19,20,21], as well as gastric, pancreatic, hepatobiliary and colorectal cancers [16,22]. Death cases in adults were also reported in association with infections by this subspecies. Thus far, infections regarding this subspecies have been noted in many countries and regions, including Mainland China, Hong Kong, Taiwan, USA, France, Amsterdam, Spain, Netherlands, Japan, Korea, etc. [9,17,21,23,24,25,26,27,28].

Macrolides are often used to treat infections caused by Gram-positive pathogens or Gram-negative cocci. They are also considered as alternative choices for patients with penicillin allergy. However, macrolide resistance has emerged in *S. gallolyticus* [22,29,30,31]. In Gram-positive bacteria, active efflux and 23S rRNA modification are two major mechanisms responsible for macrolide resistance [32,33]. Active macrolide efflux has been reported in streptococci [34,35], and it is mediated by a membrane protein encoded by the *mefA* or *mefE* gene. Alternatively, 23S rRNA modification confers macrolide-lincosamide-streptogramin B (MLS) resistance. Although this resistance can involve mutations within the L4 and L22 ribosomal proteins or 23S rRNA [36], it is generally conferred by 23S-rRNA methylating Erm enzymes. The methyl group at A2058 of 23S rRNA transferred by Erm proteins sterically disrupts the binding between macrolides and rRNA, thus rendering bacteria resistance [37]. Expression of MLS resistance in streptococci can be either constitutive or inducible [38,39]. Indeed, resistance in reported *S. gallolyticus* isolates was either caused by the presence of *ermB* gene [30], *ermT* gene [31], or to a lesser extent *mefA* gene [29,30]. While roughly 63% of clinical isolates from Taiwan were macrolide resistant [22,32], very limited information is known about the molecular mechanism of antibiotic resistance in *S. gallolyticus* subsp. *pasteurianus*. Thus far, the only mechanistic characterization of antibiotic resistance in this subspecies indicated that the presence of *ermT* [32] is responsible for macrolide resistance in 60 isolates from Taiwan. To further our understanding and facilitate clinical treatment of infections associated with this subspecies, this study aims to identify the molecular mechanism underlying the high macrolide resistance in the six isolates of *S. gallolyticus* subsp. *pasteurianus* from China.

## 2. Results

### 2.1. All S. gallolyticus subsp. pasteurianus Isolates Were Multi-Drug Resistant, with High Level Macrolide Resistance

All six isolates were identified as *S. gallolyticus* subsp. *pasteurianus* using biochemical characterization and 16S rRNA gene sequencing (Supplementary materials, Table S1, Figure S1). Using the agar dilution method recommended by CLSI [40,41], the MICs of these isolates were determined using ATCC 43144 and ATCC 29213 as controls. MICs of penicillin, cefotaxime and vancomycin for all isolates were smaller than individual breakpoints [40], indicating susceptible or intermediate category of all six isolates to these antibiotics (Table 1). While some isolates demonstrated resistance to chloramphenicol (2/6), levofloxacin (4/6) and gentamicin (3/6), all isolates were resistant to tetracycline (MIC = 25 mg/L (2/6), or >128 mg/L (4/6)), and to lincomycin (MIC ≥ 128 mg/L). Interestingly, all isolates were highly resistant to both erythromycin (MIC ≥ 1024 mg/L) and clarithromycin (MIC = 512 mg/L) (Table 1). The data indicated that all *S. gallolyticus* subsp. *pasteurianus* isolates were multi-drug resistant (MDR) and exhibited high macrolide resistance.

### 2.2. The High Macrolide Resistance Was neither Mainly Caused by Efflux Pump nor by Mutations within L4, L22, or 23S rRNA

In order to identify the molecular mechanism of high macrolide resistance in these isolates, we first tried to isolate plasmid DNA from these isolates. Interestingly, no plasmid was extracted from the isolates. PCR was then done to amplify known macrolide resistance determinants from genomic DNAs. The results revealed the absence of *mefA* and *mefE* genes (Table S2). This conclusion was also confirmed by whole genome sequencing of AL101002 (Genbank accession number LJPL00000000). These data demonstrated that *mefA* and *mefE* efflux pumps did not account for the high macrolide resistance in these isolates.

Since *mefA* and *mefE* efflux pumps were not the cause of high macrolide resistance, we further tested if L4 and L22 ribosomal proteins or 23S rRNA carried any mutations. The *rplD* and *rplV* genes encoding ribosomal proteins L4 and L22, respectively, were PCR-amplified and sequenced. In all isolates, sequence analysis did not reveal any nucleotide substitutions within these two genes, using *rplD* and *rplV* sequences from macrolide-susceptible ATCC 43144 as references. Additional PCRs to amplify 23S rRNA gene followed by DNA sequencing revealed nucleotide substitutions within 23S rRNA in all isolates. Taken together, these mutations included U2828C, G1355U, A1384G, A1490G and C2880U according to *E. coli* 23S rRNA numbering. Nevertheless, these mutations only produced MICs ≤ 32 mg/L (Table 2), as tested in BL21DE3 carrying individual mutant 23S rRNA. The MIC values were much lower than those observed in the six isolates (Table 1). Thus, neither ribosomal protein mutations in L4 or L22, nor nucleotide changes in 23S rRNA were mainly responsible for high resistance to macrolides in these isolates.

### 2.3. The ermB and ermT Genes Were Integrated into All Isolate Genomes

As efflux or mutations within L4, L22 or 23S rRNA did not play a major role for the high macrolide resistance of the six isolates, we then attempted to amplify *erm* genes responsible for 23S rRNA methylation, including *ermA*, *ermB*, *ermC*, *ermT* and *ermTR*. Among these *erm* genes, fragments matching only *ermB* and *ermT* were detected in the genomes of all isolates and confirmed by DNA sequencing (Table S2). PCR also detected the presence of *tetM* and *tetL* genes in all isolates (Table S2), which may explain the observed tetracycline resistance in these isolates (Table 1).

**Figure 1:**
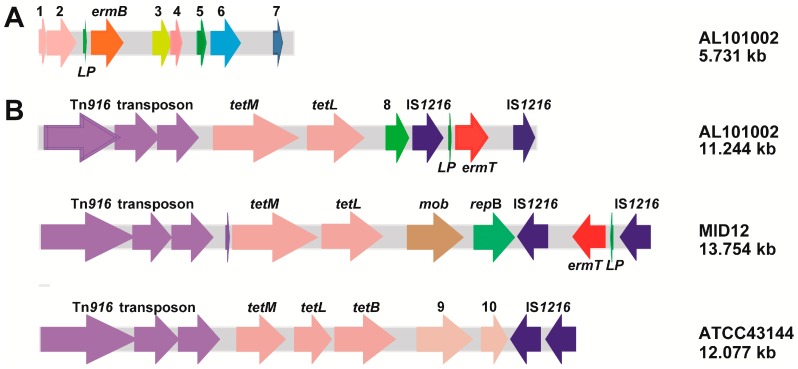
Resistance gene clusters in AL101002: (**A**) A 5.731-kb fragment harboring *ermB*. It is composed of: ABC-type transporter (1 and 2); *ermB*, putative RNA polymerase σ factor (3); Tn*916*-like transposon (4); excisionase (5); and integrase (6). Arrow 7 indicates a fragment (nt 799772–800524) of ATCC 43144 genomic DNA (Genbank accession number AP012054); (**B**) An 11.244-kb resistance gene cluster. It consists of Tn*916*, *tetM*, *tetL*, *mob*, *repB*, and IS*1216-ermT-*IS*1216* sandwich sequence. The numbers 8, 9 and 10 designate a plasmid replication protein, a plasmid recombination enzyme and a truncated plasmid replication initiation protein, respectively.

To further validate the presence of *ermB* and *ermT* genes and to elucidate the composition of the resistance gene cluster, whole genome sequencing (WGS) was performed for AL101002, a previously characterized subspecies [5]. The WGS yielded 23 contigs (Genbank accession number LJPL00000000). The 16S rDNA resides in Contig 15 and matches exactly with that from ATCC 43144, further supporting species identification in present study (Supplementary materials, Table S1, Figure S1). The flanking sequences of *ermB* and *ermT* were further identified by DNA sequencing in combination with cloning of PCR fragments. The sequencing data revealed two antibiotic resistance gene clusters of 5.731 and 11.244 kb (Genbank accession number KU511281 and KU511280), respectively. The 5′ portion of the 5.731 kb fragment encodes an ABC-type transporter, a putative conjugative transposon protein, and a leader peptide, followed by *ermB*. Downstream of *ermB*, it consisted of genes for a putative RNA polymerase σ factor, Tn*916*-like excisionase and integrase, along with a truncated α/β superfamily hydrolase (Figure 1A). This fragment shared 98% nucleotide identity with one fragment from *Clostridium difficile* 630 (accession number AM180355), a human pathogen with MDR, suggesting the possibility in exchanging resistance elements between these two types of pathogens.

The 11.244 kb fragment harbored the *ermT* gene sandwiched by a 3′ portion of IS*1216* insertion sequence (Figure 1B). Interestingly, a leader peptide was also located upstream of *ermT*. Within the upstream region a long fragment carried the Tn*916* transposon followed by *tetM* and *tetL*. In addition, a plasmid replication gene was found between *tetL* and the upstream IS*1216*. This resistance gene cluster exhibited 99% and 98% nucleotide identity with part of uncultured bacterium MID12 (accession number GU951538) and human isolate ATCC 43144 genome (accession number AP012054), respectively. The sequence identity was mainly composed of Tn*916* elements and tetracycline resistance, in addition to genes involved in plasmid replication. Variation was noted in the downstream portion of the fragment. While macrolide resistance genes were not found in ATCC 43144, *ermT* was integrated into this fragment in AL101002. This is similar to MID12, yet the sandwich sequence containing *ermT* and IS*1216* elements in AL101002 were reversed as compared with that in MID12 (Figure 1B).

### 2.4. Both ermB and ermT Were Expressed in the Isolates, and the Expression Was Erythromycin-Inducible

**Figure 2:**
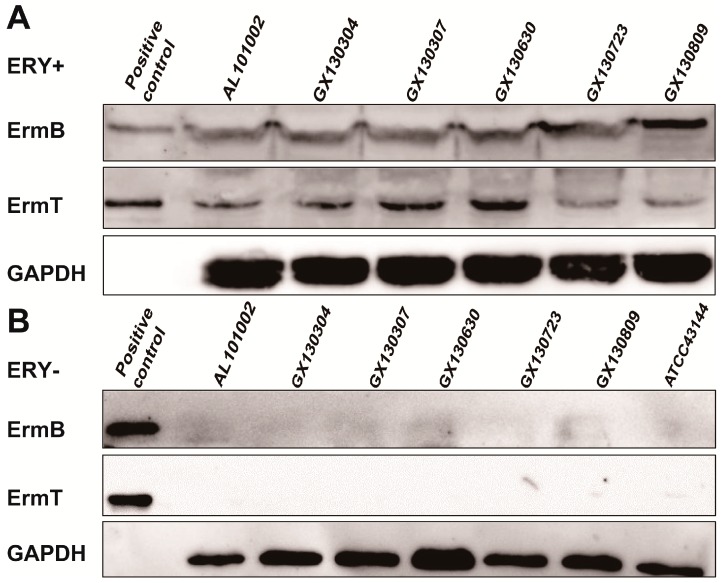
Erythromycin-inducible expression of *ermB* and *ermT*: (**A**) ErmB and ErmT protein expression in six isolates with erythromycin; and (**B**) dormancy of *ermB* and *ermT* genes in six isolates without erythromycin. Positive controls are purified recombinant ErmB and ErmT proteins tagged with a His6 tail. ErmB and ErmT bands were blotted with mouse serum against above recombinant ErmB or ErmT proteins. GAPDH was used for loading controls and was visualized by monoclonal human GAPDH antibody. ATCC 43144 is a negative control as it lacks *ermB* and *ermT* genes.

As genes in a genome may not be expressed, we further tested whether *ermB* and *ermT* genes in these isolates were expressed by Western blot (Figure 2), using in-house generated mouse serum against purified recombinant ErmB or ErmT proteins (Supplementary materials). With erythromycin, both *ermB* and *ermT* were expressed in all six isolates (Figure 2A), while without erythromycin, no ErmB or ErmT protein was detected (Figure 2B). The faint band without erythromycin could be due to non-specific recognition, as it also showed up in ATCC 43144, which does not contain any *erm* genes [31]. Additional control experiments did not reveal cross recognition of the serums for the two recombinant proteins. The data suggested that the expression of the two genes was erythromycin-inducible.

Due to immature genetic manipulation system in *S. gallolyticus* subsp. *pasteurianus*, we cloned *ermB* and *ermT* with the leader peptide into pET21a vector (Figure S2). These clones were used to test the expression of the two genes. In BL21DE3 cells, the two genes carried in pET21a were also expressed in the presence of erythromycin, as blotted by monoclonal anti-Histag antibody (Figure S2). This is in agreement with the inducible expression of the two genes in six isolates (Figure 2). The expressed ErmB and ErmT proteins without erythromycin could be due to leaky expression off the *lac* promoter in pET21a vector.

### 2.5. The Inducible Expression of ermB and ermT Conferred High Level of Macrolide Resistance

All *ermB* and *ermT* clones with or without the leader peptide in pET28a or PHT01 were transformed into BL21DE3 cells for antibiotic sensitivity testing (Table 3). MIC values for erythromycin and clarithromycin were recorded with 30 μg/mL PAβN, an efflux inhibitor [42]. While empty vectors generated an MIC of 1 mg/L for both erythromycin and clarithromycin, both *ermB* and *ermT* plasmids yielded high macrolide resistance, with an MIC of 1024 mg/L for erythromycin, and ≥128 mg/L for clarithromycin. These plasmids also exhibited high resistance to lincomycin (MIC = 512 mg/L). Interestingly, when both plasmids were introduced, MICs increased by at least two-fold for erythromycin, and to an even larger extent for clarithromycin. Similar pattern was observed for *ermBL* and *ermTL*, the clones with the leader peptide. Notably, the leader peptide appeared to reduce MIC values (Table 3). The MIC levels produced by both *ermBL* and *ermTL* plasmids were comparable to that obtained from the six *S. gallolyticus* subsp. *pasteurianus* isolates (Table 1), indicating that the presence of both *ermB* and *ermT* genes was responsible for the high macrolide resistance in these isolates.

## 3. Discussion

In this report, we have demonstrated that the inducible expression of both *ermB* and *ermT* confers high macrolide resistance in multidrug resistant *S. gallolyticus* subsp. *pasteurianus* from China.

All six isolates exhibited no resistance to penicillin, cefataxime or vancomycin, but to tetracyclin, lincomycin, arithromycin and clarithromycin among tested antibiotics. Only some isolates demonstrated resistance to chloramphenicol, levoflaxacin and gentamycin (Table 1). Limited by small number of isolates, we did not further characterize the molecular mechanism underlying the resistance to chloramphenicol, levoflaxacin and gentamycin. The tetracyclin resistance in all our isolates appeared to be conferred by *tetM* and *tetL*, as indicated by PCR amplification followed by DNA sequencing (Table S2). As lincomycin and macrolides share similar mechanism of action and MLS resistance element including *erm* [38], the lincomycin resistance in our isolates is likely contributed by the presencce of both *ermB* and *ermT* (Table 1 and Table 3). Detailed mechanisms remain to be addressed for tetracyclin and lincomycin.

While 63% of macrolide resistance was observed among *S. gallolyticus* subsp. *pasteurianus* isolates from human clinic in Taiwan [22,32], all our isolates exhibited high macrolide resistance, and acquired *ermB* as additional macrolide-resistant determinant.

Although macrolide resistance in Gram-positive bacteria is mainly contributed by active efflux and 23S rRNA modification [32,33], the high macrolide resistance in our isolates was not caused by efflux pump (Table S2). 23S rRNA modification can originate from mutations within L4, L22 and 23S rRNA or from Erm methylases [36,43]. Our study demonstrated that neither L4, L22 nor 23S mutation but the presence of both *ermB* and *ermT* (Table 1, Table 2 and Table 3) was responsible for high macrolide resistance in these isolates. The *ermB* and *ermT* genes appeared to be acquired independently (Figure 1), and their expression in all six isolates was erythromycin inducible (Figure 2).

To our knowledge, this is the first detailed mechanistic report on the presence of *ermB* and *ermT* conferring high macrolide resistance in bacteria. Previously, the presence of *ermB* and *ermT* has been reported in one single *S. bovis* isolate from Hong Kong [44] and one single group B *Streptococcus* [45], while detailed mechanism was not addressed in either report. Our data revealed not only the presence of both *ermB* and *ermT*, but also the erythromycin-inducible expression of the two genes conferring high macrolide resistance in all six isolates. Erm proteins including ErmB and ErmT are believed to methylate A2058 of 23S rRNA [46]. The acquisition of both *ermB* and *ermT* in bacterial genome is intriguing. MIC testing data using *ermB* and *ermT* clones indicated that the leader peptide led to reduced MIC values for all tested macrolides (Table 3), suggesting reduced expression of both ErmB and ErmT in the presence of the leader peptide. The leader peptide of *erm* genes is known to cause translation attenuation, and expose the open reading frame of downstream *erm* gene, thus turning on its expression [47,48]. The ribosome stalls between the 10th amino acid and the 11th incoming aminoacyl-tRNA. The translational pause largely relies on the sequence content of the leader peptide [49,50]. The reduced expression of a single *erm* gene with an upstream leader peptide in our isolates may not sustain macrolide resistance pressure. This may favor the emergence of second *erm* gene in these bacteria. The inducible expression of both *ermB* and *ermT* with an upstream leader peptide may be able to generate sufficient methylation at A2058 of 23S rRNA, thus excluding macrolides from the ribosomal polypeptide exit tunnel more efficiently than either enzyme alone, leading to enhanced macrolide resistance in host cells (Table 3). The detailed ribosomal methylation mechanisms and expression pattern of these two Erm proteins still remain to be addressed.

In summary, the present study reveals new features of macrolide resistance in *S. gallolyticus* subsp. *pasteurianus*, in which the inducible expression of both *ermB* and *ermT* conferred high macrolide resistance.

## 4. Materials and Methods

### 4.1. Bacterial Isolates and Culture Conditions

Six isolates of *S. gallolyticus* subsp. *pasteurianus* were obtained from dead ducklings collected in Hubei and Guangxi in China during several natural outbreaks in 2010–2013. The isolates were identified using biochemical characterization and 16S rRNA gene sequencing [51] (Table S1). *S. gallolyticus* subsp. *pasteurianus* ATCC 43144 and *S. aureus* ATCC 29213 were used as controls [31,52]. The *S. gallolyticus* subsp. *pasteurianus* were routinely cultured at 37 °C in trypticase soy broth (TSB) supplemented with 5% fetal bovine serum. *E. coli* TG1 and BL21DE3 were cultured in LB medium at 37 °C, and used for gene cloning and recombinant protein expression, respectively. Materials and reagents were purchased from Sigma Aldrich (Shanghai, China), unless otherwise stated.

### 4.2. Antimicrobial Susceptibility Testing

The standard agar dilution method recommended by CLSI [40,41] was used to determine MICs of various antibiotics for the six isolates. The resistance contribution by *ermB* and *ermT* genes and by 23S rRNA mutations was tested in 96-well plates using BL21DE3 cells. Each well contained 200 μL of Mueller-Hinton (MH) medium with 1000 log-phase CFUs and 30 μg/mL Phenylalanine-Arginine β-Naphthylamide (PAβN) [42]. Then, 1 mM IPTG was added to cells carrying *ermB* and/or *ermT* plasmid(s) to induce protein expression but not to cells with mutant 23S rRNA. Next, 50 μg/mL kanamycin was added to clones in pET28a, while 100 μg/mL ampicillin was used for clones in PHT01 or PUC19. Finally, the culture was incubated at 37 °C for 20 h. The cell density was determined by OD600 using a μQuant plate reader (BioTek, Beijing, China).

### 4.3. Sequence Analysis and Molecular Cloning

Genomic DNA of six isolates was extracted using TIANamp Bacteria genomic DNA kit from Tiangen Biotech (Beijing, China). PCR followed by DNA sequencing was used to detect the presence of antibiotic resistance genes and mutations within *rplD*, *rplV* and 23S rRNA gene. All PCRs were done using Phu DNA polymerase (Thermo Scientific, Waltham, MA, USA) and primers synthesized by Sangon Biotech (Shanghai, China) (Table S3). Sequence alignment was done using BLAST (NCBI web) or Clustal at http://www.ebi.ac.uk/tools/clustalw2. *ermB* and *ermT* sequences from Genbank (accession number JN899586 and AY894138) were referenced, while 23S rRNA gene, *rplD* and *rplV* sequences from ATCC 43144 (accession number AP012054) were used for detecting mutations in all isolates.

The *ermB* and *ermT* genes with or without the leader peptide from GX130630 were cloned into pET28a via NheI and XhoI sites or pET21a via NheI and NdeI sites, or into PHT01 via BamHI and XbaI sites. The 23S rRNA gene sequence from ATCC 43144 was cloned into PUC19 vector via BamHI and EcoRI sites. All clones were confirmed by DNA sequencing.

The flanking sequences of *ermT* were identified by whole genome sequencing, while those of *ermB* were determined by DNA sequencing of cloned PCR fragments. Briefly, AL101002 genomic DNA was digested by HindIII. The resulting DNA fragments were amplified and cloned using LA-PCR in vitro clone kit from Takara. Primers C1 and C2 were provided by manufacturer, while gene-specific primers were designed according to *ermB* sequence and were from Sangon Biotech, China (Table S3).

### 4.4. Whole-Genome Sequencing

The whole genome of *S. gallolyticus* subsp. *pasteurianus* AL101002 was sequenced using the Illumina HiSeq 2500 platform at Shanghai Bohao Biotechnology, China. The Whole Genome Shotgun sequence of AL101002 was de-novo assembled using SPAdes 2.5.1 (Algorithmic Biology Lab, St Petersburg, Russia) (http://bioinf.spbau.ru/spades) and has been deposited at GenBank under the accession number LJPL00000000.

### 4.5. Western Blot Analysis

The ErmB and ErmT levels in six isolates were detected by immunoblotting. The cells were cultured in TSB medium at 37 °C to early stationary phase. The cell pellet was collected after centrifugation and washed. After one freeze-thaw cycle, total protein was extracted using the Bacterial Protein Extraction Kit from Jiangsu KeyGEN BioTECH, Nanjing, China. SDS-PAGE and blotting were performed using Bio-Rad electrophoresis and blotting systems (Bio-Rad, Wuhan, China). The ErmB and ErmT proteins were detected using in-house mouse serum (1:2000) raised against recombinant ErmB or ErmT protein. Glyceraldehyde-3-phosphate dehydrogenase (GAPDH) was detected using monoclonal antibody against human GAPDH (1:5000) from Proteintech (Wuhan, China). Monoclonal goat anti-mouse IgG-HRP (1:2000) from Wuhan Boster (Wuhan, China) was used as a secondary antibody. Enhanced chemiluminescence (ECL) substrate from Seven Sea Biotech (Shanghai, China) was used for visualization. Digital images were taken using the ImageQuant LAS 4000 instrument from GE Healthcare (Beijing, China).

## Figures and Tables

**Table 1:** Minimum inhibitory concentrations of six *S. gallolyticus* subsp. *pasteurianus* isolates.

Antibiotics	MIC (mg/L) of Subspecies							
	AL 101002	GX 130304	GX 130307	GX 130630	GX 130723	GX 130809	ATCC 43144	ATCC 29213
Erythromycin	1024	1280	1280	1280	1280	1024	<0.25	0.25
Clarithromycin	512	512	512	512	512	512	<0.25	0.25
Lincomycin	128	>256	>256	256	128	256	0.5	0.5
Tetracyclin	25	128	128	25	256	256	1	0.5
Chloramphenicol	2	32	32	2	2	2	2	2
Levofloxacin	8	8	4	8	4	8	4	0.25
Gentamycin	1	8	8	1	512	0.5	8	0.5
Penicillin	0.25	0.25	0.25	0.25	0.25	0.25	0.25	2
Cefotaxime	0.25	0.25	0.25	0.25	0.25	0.125	<0.0625	2
Vancomycin	<1	<1	<1	<1	<1	<1	<1	2

**Table 2:** MICs of macrolides contributed by nucleotide substitutions in 23S rRNA.

Mutation in 23S rRNA		Found in Isolates	Location in 23S rRNA	MIC (mg/L)	
*S. pasteurianus* Numbering	*E. coli* Numbering			Erythromycin	Clarithromycin
Wild Type		ATCC 43144		1	1
U2824C	U2828C	AL101002	Domain VI	1	4
		GX130307			
		GX130630			
		GX130809			
C2876U	C2880U	GX130723	Domain VI	2	8
U2824C + C2876U	U2828C + C2880U	GX130723	Domain VI	4	16
G1380U + A1409G + A1515G	G1355U + A1384G + A1490G	GX130304	Domain III	32	32

**Table 3:** MICs of *ermB* and *ermT* clones. *ermTL* and *ermBL* represent the *ermT* and *ermB* clones with upstream leader peptide.

*erm* Genes and Plasmids	MICs (mg/L)		
	Erythromycin	Clarithromycin	Lincomycin
pET28a	1	1	128
PHT01	1	1	128
*ermT* in pET28a	1024	512	512
*ermTL* in pET28a	512	256	512
*ermB* in pET28a	1024	128	512
*ermBL* in pET28a	512	8	512
*ermT* in PHT01	128	16	–
*ermTL* in PHT01	16	4	–
*ermB* in pET28a + *ermT* in PHT01	2048	>1024	2560
*ermBL* in pET28a + *ermTL* in PHT01	1024	1024	2048

## Supplementary Materials

Supplementary materials can be found at www.mdpi.com/1422-0067/17/10/1599/s1.

## Abbreviations

MIC	Minimum inhibitory concentration
ATCC	American type culture collection
CLSI	Clinical and laboratory standards institute
MLS	Macrolide-lincosamide-streptogramin B
TSB	Trypticase soy broth
MH	Mueller-hinton
IPTG	Isopropyl β-d-1-thiogalactopyranoside
ECL	Enhanced chemiluminescence
GAPDH	Glyceraldehyde-3-phosphate dehydrogenase
PAβN	Phenylalanine-arginine beta-naphthylamide
