# Supplementary material for: Inducible Expression of both ermB and ermT Conferred High Macrolide Resistance in Streptococcus gallolyticus subsp. pasteurianus Isolates in China
Source: Int J Mol Sci. 2016 Sep 22;17(10):1599. doi: 10.3390/ijms17101599 (PMC5085632; doi:10.3390/ijms17101599)
Supplement: Supplementary file 1 [file ijms-17-01599-s001.pdf]

Supplementary Materials: Inducible Expression of both *ermB* and *ermT* Conferred High Macrolide Resistance in *Streptococcus gallolyticus* subsp. *pasteurianus* Isolates in China

Meixia Li, Chao Cai, Juan Chen, Changwei Cheng, Guofu Cheng, Xueying Hu and Cuiping Liu

1. Results and Discussion

1.1. All Six Isolates Are S. gallolyticus subsp. pasteurianus

Phenotypic characterization for all isolates revealed biochemical features of *S. gallolyticus* subsp. *pasteurianus* [1,2] (Table S1). The amplified 16S rRNA gene fragment using genomic DNA from all six isolates (Figure S1) exhibited 100% sequence identity with that from *S. gallolyticus* subsp. *pasteurianus* ATCC43144 (data not shown). These data demonstrated that all six isolates are *S. gallolyticus* subsp. *pasteurianus*. WGS data of AL101002 further supported the strain identification of this study.

Table S1. Phenotypic characteristics of six isolates.

Phenotype		AL 101002	GX 130304	GX 130307	GX 130630	GX 130723	GX 130809	*S. gallolyticus* subsp. *pasteurianus*
**Hydrolysis**	Aesculin	+	+	+	+	+	+	+
**Production of**	β-Glucosidase	+	+	+	+	+	+	+
	β-Glucuronidase	−	−	−	−	−	−	+
	α-Galactosidase	+	+	+	+	+	+	V
	β-Galactosidase	−	−	−	−	−	−	−
	β-Mannosidase	−	−	−	−	−	−	+
**Acidification of**	Glycogen	−	−	−	−	−	−	−
	Lactose	+	+	+	+	+	+	+
	Mannitol	−	−	−	−	−	−	−
	Raffinose	+	+	+	+	+	+	V
	Trehalose	+	+	+	+	+	+	+

+ refers to positive reaction; − means negative reaction. AL101002 has been previously characterized as *S. gallolyticus* subsp. *pasteurianus* [1]. Data for *S. gallolyticus* subsp. *pasteurianus* were from literature [2]. V means 21%–79% strains positive.

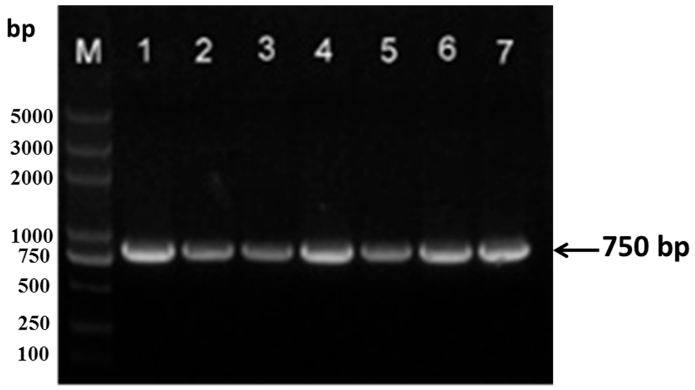

Figure S1. PCR amplification of 16S rDNA fragment from six isolates. The amplified 16S rDNA fragments of 750 bp were resolved in an agarose gel. **Lanes 1–7** were AL101002, GX130304, GX130307, GX130630, GX130723, GX130809 and ATCC43144, respectively. **Lane M** is DNA marker.

1.2. Expression of ermB and ermT in pET21a Vector

Using BL21DE3 host cells containing either *ermB* or *ermT* with leader peptide in pET21a vector, the levels of each protein were analyzed by Western blot analysis using a commercial monoclonal anti-His6 antibody. While empty pET21a vector did not yield any protein band recognizable by His6-tag antibody, the *ermB* and *ermT* clone in the pET21a vector gave a distinct band corresponding to 27 kD when erythromycin was supplemented. The expression level of *ermB* appeared to be stable with erythromycin, which was not affected by increasing erythromycin concentration. Interestingly, the *ermB* showed a very weak band when erythromycin was depleted. The *ermT* was also expressed with or without erythromycin in BL21DE3 cells. Its expression increased with higher level of erythromycin (Figure S2). The observed ErmB and ErmT protein in BL21DE3 cells supported erythromycin-inducible expression in *S. gallolyticus* subsp. *pasteurianus* isolates. The expression of the two proteins without erythromycin could be attributed by the leaky expression off the *lac* promoter in pET21a vector.

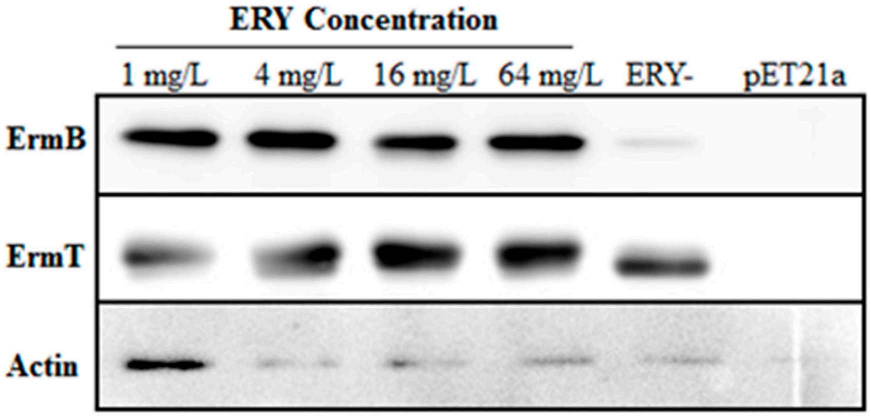

Figure S2. *ermB* and *ermT* expression in BL21DE3 cells. Cell lysates from BL21DE3 containing either *ermB* or *ermT* in pET21a with 0–64 mg/L of erythromycin were resolved on a 12% SDS-PAGE. ErmB and ErmT protein were blotted using a commercial monoclonal His6 antibody. Actin expression served as loading control. pET21a vector was a negative control. ERY stands for erythromycin.

2. Materials and Methods

2.1. Strain Identification

All isolates in this study were identified using biochemical characterization along with 16S rRNA gene sequencing [3].

2.2. Protein Expression and Purification

BL21DE3 cells harboring *ermB*- or *ermT*-containing pET28a plasmids were seeded and cultured at 37 °C overnight in LB medium supplemented with 50 μg/mL kanamycin. The overnight culture was diluted in 1L LB medium with 25 μg/mL kanamycin and grown at 37 °C to an OD_{600} within 0.4–0.6. Isopropyl β-D-1-thiogalactopyranoside (IPTG) was added into the culture to a final concentration of 0.8 mM. The culture was further incubated at 25 °C for 5 h. Cells were harvested and spun down at 16,000 rpm at 4 °C. Cell pellet was resuspended in a buffer containing 20 mM Tris-HCl, pH 8.0, 100 mM NaCl, 5 mM β-mercaptoethanol and 2 mg/mL lysozyme, and further homogenized using sonication. The cell lysate was incubated with 1.5 mL of Ni-NTA resin (Qiagen) at 4 °C under gentle agitation. The recombinant ErmB and ErmT proteins carrying a His6-tag were eluted using imidazole in above lysis buffer in a gravity column [4,5]. The fractions were analyzed using SDS-PAGE and pooled accordingly.

2.3. Preparation of Polyclonal Antibodies against ErmB and ErmT

The polyclonal antibodies were prepared following published procedures [6]. 600 μg/mL purified recombinant ErmB and ErmT proteins were separately mixed with an equal volume of Freund's complete adjuvant (Sigma-Aldrich, St. Louis, MO, USA). Subsequently, 100 μg ErmB or ErmT in the adjuvant was injected subcutaneously into 6 week-old BALAB/c mice. Two weeks after the first injection, the mice were immunized twice at 1-week interval, using same amount of each protein. Blood was collected 7 days after the last injection and placed at room temperature for 1 h, and then at 4 °C overnight. The supernatant was collected via centrifugation at 2200× *g* for 10 min. The above experimental protocols were performed according to the Regulations of the Standing Committee of Hubei People's Congress and approved by the Ethical Committee of Huazhong Agricultural University (4200695757, 29 July 2005), China.

2.4. Western Blot Analysis

Monoclonal anti-Histag (1:5000) from ProteinTech was used to detect ErmB and ErmT protein levels in BL21DE3 cells containing *ermB* or *ermT* expression clones in pET21a vector. Cells were cultured in LB medium for 8 h at 37 °C with various concentration of erythromycin. One mL of culture was taken and spun down. After removing the supernatant, cell pellet was resuspended in 1× Laemmli Sample Buffer and boiled for 5 min. 20 μg of total protein was applied to 12% SDS-PAGE. After transfer to nitrocellulose membrane, ErmB and ErmT were blotted with monoclonal Histag antibody, followed by goat anti-mouse IgG-HRP secondary antibody. Actin was used as loading control. Its expression was detected using β-actin monoclonal antibody (1:2000) from ProteinTech (Wuhan, China). The images were visualized using ECL substrate kit from Seven Sea Biotech (Shanghai, China) and taken by ImageQuant LAS 4000 instrument from GE Healthcare (Beijing, China).

Table S2. Resistance genes in six isolates.

**Resistance Gene**	**Isolates**					
	**AL101002**	**GX130304**	**GX130307**	**GX130630**	**GX130723**	**GX130809**
ermB	+	+	+	+	+	+
ermT	+	+	+	+	+	+
tetM	+	+	+	+	+	+
tetL	+	+	+	+	+	+
ermA	−	−	−	−	−	−
ermC	−	−	−	−	−	−
ermTR	−	−	−	−	−	−
mefA	−	−	−	−	−	−
mefE	−	−	−	−	−	−
Aac(6')-alpha(2')	−	−	−	−	+	−

+ indicates the presence of a resistance gene; − means the absence of a resistance gene.

Table S3. PCR Primers used in this study.

Purpose	Gene	Primer Sequence	Reference
Fragment Amplification	*16S rDNA*	5′ GAG TTT GAT CMT GGC TCA G 3′<br>5′ CTA HAG GGT ATC TAA TCC T 3′	[3]
	rplD	5′ TAA CGT ACC GGG TGC TAA G 3′<br>5′ GAT TTT TCA GTG ATT ACT GG 3′	
	rplV	5′ GTC ACA AAC TTG GTG AAT TC 3′<br>5′ AAT CCG CGT ATT CTT TTT CAG 3′	
	23S rDNA	5′ CCG GAA TTC TTA AGT TAA TAA GGG CGC AC 3′<br>5′ CGC GGA TCC TTG GAT AAG TCC TCG AGC TA 3′	
	ermA	5′ TCT AAA AAG CAT GTA AAA GAA 3′<br>5′ CTT CGA TAG TTT ATT AAT ATT AGT 3′	[7]
	ermB	5′ ATG AAC AAA AAT ATA AAA TAT TC 3′<br>5′ TTA TTT CCT CCC GTT AAA TA 3′	
	ermC	5′ TCAAAA CAT AAT ATA GAT AAA 3′<br>5′ GCT AAT ATT GTT TAA ATC GTC AAT 3′	[7]
	ermT	5′ CTA CTA GCT AGC ATG AAC AAA AAA AAT ATA AAA G 3′<br>5′ CGA CTG CTC GAG T TAT CTA TTA AAT AAT TTA TAG 3′	
	ermTR	5′ ATA GAA ATT GGG TCA GGA AAA G 3′<br>5′ TTG ATT TTT AGT AAA AAG 3′	[8]
	mefA/mefE	5′ AGT ATC ATT AAT CAC TAG TGC 3′<br>5′ TTC TTC TGG TAC TAA AAG TGG 3′	[7]
	tetM	5′ ATG GAG GAA AAT CAC ATG AA 3′<br>5′ CTA AGT TAT TTT ATT GAA CAT A 3′	
	tetL	5′ ATG GTT TTG AAC GTC TCA TTA C 3′<br>5′ TTA GAA ATC CCT TTG AGA ATG 3′	
	aac(6′)-aph(2′)	5′ CTA CTA GCT AGC ATG AAT ATA GTT GAA AAT G 3′<br>5′ CGA CTG CTC GAG TCA ATC TTT ATA AGT CCT T 3′	
	ermB	5′ CTA CTA GCT AGC ATG AAC AAA AAT ATA AAA TAT TC 3′<br>5′ CGA CTG CTC GAG TTA TTT CCT CCC GTT AAA TA 3′	
	ermT	5′ CTA CTA G′CTAGCATG AAC AAA AAA AAT ATA AAA G 3′<br>5′ CGA CTG C′TCGAGT TAT CTA TTA AAT AAT TTA TAG 3′	

Table Ss. *Cont.*

Purpose	Gene	Primer Sequence	Reference
**Clone into PHT01**	*ermT*	5′ CGC G'GATCC ATG AAC AAA AAA AAT ATA AAA G 3′<br>5′ GC TCT AGA TTA TCT ATT AAA TAA TTT ATA GC 3′	
	ermBL	5′ CGC GGA TCC ATG TTG GTA TTC CAA ATG CG 3′<br>5′ CGA CTG CTC GAG TTA TTT CCT CCC GTT AAA TA 3′	
	ermTL	5′ CGC GGA TCC ATG GGC ATT TTT AGT ATT TTT G 3′<br>5′ CGA CTG CTCGAGT TAT CTA TTA AAT AAT TTA TAG 3′	
**Clone into pET28a**	*ermBL*	5′ CGC GGA TCC ATG TTG GTA TTC CAA ATG CG 3′<br>5′ GC TCT AGA TTA TTT CCT CCC GTT AAA TAA T 3′	
	ermTL	5′ CGC GGA TCC ATG GGC ATT TTT AGT ATT TTT G 3′<br>5′ GC TCT AGA TTA TCT ATT AAA TAA TTT ATA GC 3′	
**Clone into pET21**	*ermBL*	5′ GGG AA TT C CAT ATG ATG TTG GTA TTC CAA ATG CG 3′<br>5′ CGA CTG CTCGAG TTT CCT CCC GTT AAA TA ATAG 3′	
	ermTL	5′ GGG AA TT C CATATG ATG GGC ATT TTT AGT ATT TTT G 3′<br>5′ CGA CTG CTCGAG T CTA TTA AAT AAT TTA TAG CTA 3′	
**Cloning**	C1<br>C2<br>Upstream of<br>*ermB*<br>Downstream of<br>*ermB*	5′ GTACATATTGTCGTTAGAACGCGTAATACGACTCA 3′<br>5′ CGTTAGAACGCGTAATACGACTCACTATAGGGAGA 3′<br>5′ AGA TAG ATG TCA GAC GCA CGG C 3′<br>5′ CCT GTT CCA ATT TCG TAA ACG G 3′<br>5′ TC TTG CAC ACT CAA GTC TCG 3′<br>5′ CCA TAC CAC AGA TGT TCC AG 3′	
**Amplify the sequence between *ermT* and *tet***	Upstream of<br>*ermT*<br>Downstream of *ermT*	5′ ATG AGT TAT GCA GTT TGTA GAA TGC 3′<br>5′ AAC GAA GAT AGT AGC CCA CGG 3′<br>5′ ACT AGC ACT ATT TTT AAT GAC AGA AGT TGA 3′	[9]
**Mutation in 23S rDNA in Plasmid**	C2876T	5′ ACA TGT AGC GGA CTA ATA TTA ATA GCT CGA GGA CTT ATC 3′<br>5′ GAT AAG TCC TCG AGC TAT TAA TAT TAG TCC GCT ACA TGT 3′	
	T2824C	5′ GAG CCC TGA GAG ATG ATC AGG TAG ATA GGT TAG G 3′<br>5′ C CTA ACC TAT CTA CCT GAT CAT CTC TCA GGG CTC 3′	
	A1380T	5′ GTT AGT CGG GAC CTA ATG AGA GAC CGA AAG GTG 3′<br>5′ CAC CTT TCG GTC TCT CAT TAG GTC CCG ACT AAC 3′	
	A1409G	5′ GT GTA TCC GAT GGC CGA CAG GTT GAT ATT CCTG 3′<br>5′ CAGG AAT ATC AAC CTG TCG GCC ATC GGA TAC AC 3′	
	A1515G	5′ GCA GTG AGG TGT GAT ATG GGT CAA ATG CTT GTA TCT C 3′<br>5′ G AGA TAC AAG CAT TTG ACC CAT ATC ACA CCT CAC TGC 3′	

References

1. Li, M.; Gu, C.; Zhang, W.; Li, S.; Liu, J.; Qin, C.; Su, J.; Cheng, G.; Hu, X. Isolation and characterization of *Streptococcus gallolyticus* subsp. *pasteurianus* causing meningitis in ducklings. *Vet. Microbiol.* **2013**, *162*, 930–936.
2. Schlegel, L.; Grimont, F.; Ageron, E.; Grimont, P.A.D.; Bouvet, A. Reappraisal of the taxonomy of the *Streptococcus bovis*/*Streptococcus equinus* complex and related species: Description of *Streptococcus gallolyticus* subsp. *gallolyticus* subsp. nov., *S. gallolyticus* subsp. *macedonicus* subsp. nov. and *S. gallolyticus* subsp. *pasteurianus* subsp. nov. *Int. J. Syst. Evol. Microbiol.* **2003**, *53*, 632–645.
3. Wilson, K.H.; Blitchinqton, R.; Greene, R.C. Amplification of bacterial 16S ribosomal DNA with polymerase chain reaction. *J. Clin. Microbiol.* **1990**, *28*, 1942–1946.
4. Liu, C.; Gamper, H.; Liu, H.; Barry, S.C.; Hou, Y.M. Potential for interdependent development of tRNA determinants for aminoacylation and ribosome decoding. *Nat. Commun.* **2011**, *2*, 329.
5. Liu, C.; Sanders, J.M.; Pascal, J.M.; Hou, Y.M. Adaptation to tRNA acceptor stem structure by flexible adjustment in the catalytic domain of class I tRNA synthetases. *RNA* **2012**, *18*, 213–221.
6. Laverde, D.; Wobser, D.; Romero-Saavedra, F.; Hogendorf, W.; Marel, G.V.D.; Berthold, M.; Kropec, A.; Codee, J.; Huebner, J. Synthetic teichoic acid conjugate vaccine against Nosocomial Gram-positive bacteria. *PLoS ONE* **2014**, *9*, e110953.
7. Sutcliffe, J.; Grebe, T.; Tait-Kamradt, A.; Wondrack, L. Detection of erythromycin-resistant determinants by PCR. *Antimicrob. Agents Chemother.* **1996**, *40*, 2562–2566.
8. Kataja, J.; Huovinen, P.; Skurnik, M.; Seppala, H. Erythromycin resistance genes in group A streptococci in Finland. *Antimicrob. Agents Chemother.* **1999**, *43*, 48–52.
9. Tsai, J.C.; Hsueh, P.R.; Chen, H.J.; Tseng, S.P.; Chen, P.Y.; Teng, L.J. The *erm*(T) gene is flanked by IS1216V in inducible erythromycin-resistant *Streptococcus gallolyticus* subsp. *pasteurianus*. *Antimicrob. Agents Chemother.* **2005**, *49*, 4347–4350.
